# European forestry systems mirror social-ecological diversity but closer-to-nature forest management and landscape planning are also required

**DOI:** 10.1038/s41598-026-36659-z

**Published:** 2026-01-26

**Authors:** Per Angelstam, Michael Manton, Thomas A. Nagel, Metodi Sotirov

**Affiliations:** 1 Department of Forestry and Wildlife Management, University of Inland Norway, Campus Evenstad, 2480 Koppang, Norway; 2 Bioeconomy Research Institute, Vytautas Magnus University, Studentu g. 13, Kauno r, LT-53362 Akademija, Lithuania; 3 Department of Forestry and Renewable Forest Resources, Biotechnical Faculty, University of Ljubljana, Večna Pot 83c, 1000 Ljubljana, Slovenia; 4 Chair of Forest and Environmental Policy, University of Freiburg, Tennenbacher Str. 4, 9106 Freiburg im Breisgau, Germany

**Keywords:** Sustainable forest management, Closer-to-nature forest management, Multifunctional landscape, Segregation and integration, Triad, Spatial planning, Ecology, Ecology, Environmental sciences

## Abstract

New forest-related policies lead to conflicts among different forest benefits, actors and stakeholders. This requires multiple tools for forest management and landscape planning towards multifunctional landscapes. We first explore if the relative proportions of clearcutting (CC) and continuous cover forestry (CCF) as the two major contrasting traditional forest management systems in Europe can be explained by the complex net effect of biophysical, anthropogenic and social system variables. We then review mismatches between CC and CCF methods, and the tools required by new policies advocating multifunctional forest landscapes. The first three components of a multivariate analysis (PCA) explained 66% of the variation in the dataset. There were four main clusters of countries: (1) Nordic-Baltic boreal, (2) continental temperate lowland, (3) mountain, and (4) southern and southeastern Europe. The incidence of CCF in the 26 countries was correlated to both PC1 and PC2, and a multiple regression explained 53% of the variation in applying CCF. However, key mismatches between the application of CC and CCF and policy about multifunctional landscapes include difficulties to secure biodiversity conservation and ecosystem resilience. Therefore, new “closer-to-nature” forest management systems and triad landscape planning are also necessary.

## Introduction

To meet diverse and growing societal needs for forest ecosystem goods and services sustainable forest management was formulated as a policy and management paradigm at both international and national policy levels about three decades ago^1,2^. However, societal expectations of what forests and woodlands should provide vary considerably among locations, stakeholder groups, and over time^3,4^. This clearly indicates that if multifunctional forest landscapes are desired, then one single forest management system will not suffice.

Forest management systems focusing on wood production can be divided into two main approaches to felling and regeneration within a forest management unit: concentrated or dispersed^5–12^. The first group is clearcutting (CC) of different patch sizes and with different levels of tree retention. The second group involves continuous cover forestry (CCF), ranging from selective harvesting of single trees via selection systems, to irregular shelterwood creating openings of 1000–1500 m2^2,13^. This results in different combinations of treatment unit sizes, proportions of trees removed, and harvest return intervals across forest landscapes. Both even-aged (CC) and uneven-aged (CCF) forest management systems traditionally focus on high yield wood production^4,14^. However, to maintain biodiversity, both systems require sufficient retention of old trees, dead wood, and other naturalness structures associated with unmanaged forests^15,16^, as well as functional protected area networks^17^.

The term sustainability in forest management is thought to have been coined by von Carlowitz in 1732^18^. It referred to an economic sustainability definition of sustained timber yield, which should secure the long-term supply of wood for mining and booming industries in Central Europe linked to the industrial revolution at that time^19^. This policy and management breakthrough created a management culture of even-aged forestry aimed at maximising wood production by counteracting forest degradation and deforestation due to expansion of mining, agriculture, and urban areas, and to satisfy increased demands for wood^20^. This “Normalwaldmodell” is still a foundation of maximal sustained yield forestry today that rotational management is based on^21–23^.

Continuous cover forestry (CCF) also aims primarily at sustained yield. It appeared as a competing trend in forest management^24^. Seedre et al.^25^ defined CCF as a silvicultural method involving the partial harvest of trees, which always maintains at least 30% tree cover within a stand (e.g., by basal area, volume, etc.), and at least two age classes of production trees (i.e. shelterwood systems), and thereby an uneven-aged forest structure. This definition captures the essence of CCF and excludes even-aged forestry (rotation of even-aged stands within a forest management unit), retention forestry (a combination of even-aged forestry leaving few single and groups of trees on a harvested areas). Debates about these and other types of “clearcut-free” forestry on the one hand, and even-aged forest management on the other hand are intense^26–28^.

However, both these practices of forestry are challenged by newer policy concepts^29^, such as sustainable forest management (sensu Forest Europe https://foresteurope.org/), ecosystem management^30,31^ and multifunctional landscapes^32^. This requires the balancing of ecological, economic and social aspects, as well as systems thinking and non-linearities in both ecological and social systems^32–35^.

A key challenge for both CC and CCF is to cope with the negative effects of forest management systems aimed at maximising wood production on the maintenance of biodiversity, i.e., species, their habitat, and the processes that maintain them, as well as ecological resilience to support adaptation to climate change^4^. The new EU Biodiversity Strategy for 2030^36^ and the nature restoration regulation^37^ thus stress the need for the recovery of Europe’s biodiversity. The strategy highlights the importance of nature-based solutions supporting climate adaptation and calls for the development and application of novel forest management practices. Inspired by research about natural forest disturbance regimes^4,38,39^ the European Commission developed guidelines on closer-to-nature forest management and biodiversity friendly afforestation, reforestation and tree planting, thus stressing the need for encouraging a new environmentally centred direction of forest policy and management development^35^. The new EU Forest Strategy for 2030^32^ is also a response to this commitment and proposes “*closer-to-nature forest management as a set of practices to ensure multifunctional forests by combining biodiversity goals*,* carbon stock preservation and timber-related revenues*”.

At the same time, forest management practices in Europe mirror different biogeographical socio-economic and political-institutional contexts^40,41^. Beyond this general observation, limited systematic and specific explanations for this diversity of forest management approaches in Europe has been provided by previous research^42^. A wide range of drivers influence decisions about how to manage a specific forest area for different forest benefits^43^. Current forest-related policies of relevance for sustainable forest landscapes imply application of a social-ecological perspective, which requires inclusion of much more than traditional forest planning data like site type, tree species and volume growth. This calls for a strongly improved information base about forests and forest landscapes^44^. Focusing on three dimensions of the landscape concept as an approach to describe social-ecological systems, data should represent biophysical, anthropogenic and social system dimensions^45^.

To improve the understanding of drivers behind the application of different forest management systems in Europe and their impacts, this study has two aims. First, in a quantitative results section, we test the hypothesis that the application of forest management systems based on CC and CCF, the two most widespread contrasting systems, can be explained by a complex suite of social-ecological factors representing biophysical, anthropogenic and social systems variables. Second, in a qualitative mini-review section three topics are treated: (1) we discuss the regional distribution of CC and CCF. Next, noting the failure of current CC and CCF forest management systems to realise new sustainable forest management policy aspects like biodiversity conservation and resilience, we (2) stress the need for closer-to-nature forest management approaches that emulate disturbance regimes found in naturally dynamic forest, and in traditional cultural woodland landscapes. To succeed with this, we (3) highlight the importance of considering rotation length, the age variation of trees within and among stands in landscapes and regions, tree species composition, size of treatment area, and harvesting intensity. The final general discussion stresses the need to combine traditional CC and CCF forest management systems, and new closer-to-nature forest management systems, as well as the potential of landscape planning supporting zoning systems (e.g., triad) to meet varied demands on forests.

## Methodology

### Quantitative and qualitative analyses, and a synthesis

This study connects two aims employing two different research approaches. The first aim focuses on factors explaining the distribution of CC and CCF forest management systems in different European regions. To explain this, we employ exploratory quantitative analysis using data and statistics. The second aim is to discuss qualitative mismatches between CC and CCF management focusing on wood production on the one hand, and tools required for conserving biodiversity and resilience as components of multifunctional landscapes on the other. The final general discussion focuses on how traditional and new approaches to forest management, and landscape planning supporting zoning, can be integrated.

### Main geographical unit of analysis

**Fig. 1:**
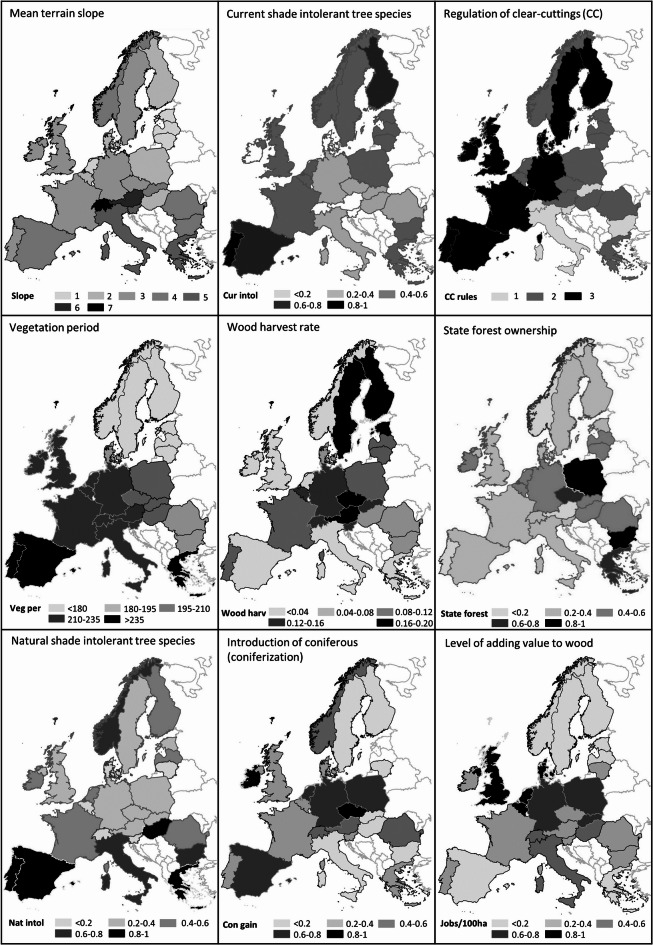
Maps showing raw not standardized parameter values of nine indicators matching biophysical, anthropogenic and social system dimensions (see Table 1). The maps were created using ArcGIS PRO 3.4.0 (https://www.esri.com/en-us/arcgis/products/arcgis-pro/overview).

Inspired by Mason’s et al.^42^ review of the distribution of continuous cover forestry (CCF) in Europe, we selected 26 countries as units for analysis to test the hypothesis that the variation in application of CC and CCF forest management depends on the portfolios of biophysical, anthropogenic and social system variables. Due to a shortage of data, we omitted Ukraine and Croatia, which were included by Mason et al.^42^, and instead included Austria and Bulgaria (Fig. 1).

## Quantitative analysis

The forests and woodlands of Europe deliver multiple benefits supporting human well-being and welfare^4,46,47^. Forest landscapes being complex social-ecological systems, we selected three groups of indicators representing biophysical, anthropogenic and social system variables. Ecosystem patterns and processes are key biophysical drivers forming natural forest landscapes^48^. Such drivers create a diversity of plant communities with different dynamics, which then form habitat networks for different species and assemblages of species. However, over millennia humans have used and thus transformed the natural vegetation. This means that the anthropogenic footprint, including both disturbances and structures at multiples scales, must be considered. Finally, the social system plays an integral role both through trajectories of historical developments, setting the policy-institutional rules and regulations for forest landscape management^49^. For each group, we selected three indicator variables (Table 1), and compiled data (Table 2).

### Variables

#### Biophysical variables

##### Mean terrain slope

Biophysical factors like the slope of forest land affect the accessibility to forest management. The more forests are placed on steep slopes, the less feasible it is to access these forests and harvest wood. Hence, the likelihood of forests being impacted by forestry is then lower^50^. This biophysical aspect may require the maintenance of protective forest functions, for example by focusing on harvesting methods that maintain forest cover on steep slopes^42^. Therefore, we used mean terrain slope as an indicator of natural forests^51^; see Tables 1 and 2.

##### Vegetation period

The geographical distribution of tree species, and the composition and structure of forests, are highly dependent on climatic conditions. The growing season (e.g., the number of days > 5 °C) is often used as an indicator for the time that trees produce biomass during a given year^52^. The rationale is that the growing season is a natural indicator of tree growth and thus the opportunity for high wood yield and opportunity for high profitability. Heat can also limit tree growth and survival^53^. We calculated the country mean growing vegetation season length as an indicator variable using phenology data from the European Commission^54^; see Tables 1 and 2.

##### Natural proportion of shade-intolerant tree species

The life history traits of trees are defined by a set of complex adaptive relationships. Large-scale biogeographic and climatic factors are connected to the regional distribution of shade-tolerant vs. shade-intolerant tree species^55^, and thus to the opportunity to favour silvicultural systems providing different light conditions. To map the natural geographical distribution of shade-intolerant tree species (e.g., *Pinus spp*.), we analysed the potential natural vegetation (PNV) data compiled by Bohn et al.^56^. Because shade-tolerance and shade-intolerance are significantly negatively correlated (*r* = −0.57), either variable can be used. The PNV data includes short descriptions and maps of all potential vegetation communities for the European continent; see Tables 1 and 2.

#### Anthropogenic variables

##### Current proportion of shade-intolerant tree species

The deliberate transformation of tree species composition found in naturally dynamic forests to effective biomass crop production systems involves favouring desired tree species. Different forest management systems create different light conditions within forest stands, which need to match the ecology of tree species in focus for management^57^. To map the current geographical distribution of shade-intolerant (e.g., *Pinus spp*.) vs. shade-tolerant tree species (e.g., the genus *Fagus spp.*,* Picea spp.*,* Abies spp.*) we analysed the data compiled by Brus et al.^58^; see Tables 1 and 2. While CC matches the former, CCF matches the latter.

##### Wood harvest

Forest management typically focuses on high sustained yield of wood and biomass, which is used for value-added production. This means that management activities focus on applying silvicultural treatments to increase the amount of wood that can be harvested annually. This implies higher forest management intensity. We used wood harvest data^59,60^ as a variable to indicate the intensity of forest management; see Tables 1 and 2.

##### Introduction of conifers

Commonly, forest management for high yields of wood has focused heavily on promoting faster-growing coniferous species (e.g., Norway spruce or pines *(Pinus spp.*) depending on region) using even-aged rotation forestry, which is combined with production of added value in forest industry. Conifer forests’ relatively simple management approach yielding high wood production with short rotation periods, and matching favourably market demands, has developed a “conifer plantation culture” among forest managers in many regions^61^. This has led to loss of deciduous forests^62^, which now has large negative implications for biodiversity and ecosystem services^63^. This issue applies also to other fast-growing tree species^64^. To measure the level of “coniferization” in Europe, we overlaid the data of the potential natural vegetation (PNV) compiled by Bohn et al.^56^ with the current distribution of tree species^58^; see Tables 1 and 2. Plantation forestry focuses on fast-growing tree species such as eucalypts and poplars.

**Table 1:** Overview of nine variables representing three themes mirroring different dimensions of forest landscapes.

Type of system	Variables	Unit	Code	References
Biophysical	Mean terrain slope Vegetation period Natural shade-intolerant tree species	Likert scale Number of days with temperature > 5 degrees C Proportion	Slope Veg per Nat intol	Milevski and Andreeska^51^ European Commission^54^ Bohn et al.^56^
Anthropogenic	Current shade-intolerant tree species Wood harvest rate Introduction of conifers (coniferization)	Proportion m^3^ ha^− 1^ yr-^1^ Proportion	Cur intol Wood harv Con gain	Brus et al.^58^ Verkerk et al.^59,60^ Brus et al.^58^ Bohn et al.^56^
Social	Regulation of clearcutting (CC) Land ownership being public or private Level of adding value to wood	Likert scale Proportion Jobs per forest area	CC rules State forest Jobs/100 ha	Sotirov et al.^41^ Pulla et al.^69^, UNECE^70^ Robert et al.^72^, Forests Europe^73^

**Table 2:** Raw data for three groups of variables representing biophysical, anthropogenic and social indicators. Sources for the nine variables are quoted in Table 1.

		Biophysical variables			Anthropogenic variables			Social variables		
Country	ID	Slope	Veg per	Nat intol	Cur intol	Wood harv	Con gain	CC rules	State forest	Jobs / 100 ha
Austria	A	6	213	0.21	0.13	0.20	0.70	2	0.18	2.82
Belgium	B	2	218	0.19	0.46	0.15	0.99	2	0.48	11.13
Bulgaria	BG	4	185	0.78	0.51	0.05	0.82	1	0.88	2.01
Czechia	CZ	3	198	0.29	0.24	0.20	0.98	2	0.77	2.81
Denmark	DK	2	184	0.16	0.12	0.06	1.00	3	0.37	8.50
Estonia	EST	1	169	0.29	0.60	0.17	0.11	2	0.36	1.97
Finland	FIN	2	164	0.40	0.61	0.17	0.13	3	0.3	0.48
France	F	3	226	0.43	0.47	0.10	0.96	3	0.24	2.04
Germany	D	3	215	0.21	0.33	0.15	0.98	3	0.52	6.21
Greece	GR	5	244	0.82	0.44	0.01	0.88	2	0.63	1.13
Hungary	H	2	205	0.87	0.28	0.06	1.00	2	0.56	5.80
Ireland	IRL	3	235	0.43	0.14	0.04	1.00	3	0.53	3.45
Italy	I	5	225	0.74	0.31	0.03	0.85	1	0.35	4.73
Latvia	LV	1	173	0.43	0.56	0.09	0.01	2	0.54	1.61
Lithuania	LT	1	179	0.20	0.48	0.09	0.24	2	0.5	2.84
Netherlands	NL	1	235	0.57	0.57	0.03	1.00	2	0.49	22.87
Norway	N	4	167	0.64	0.49	0.03	0.67	2	0.12	0.21
Poland	PL	2	197	0.36	0.55	0.10	0.90	2	0.82	6.20
Portugal	PT	3	243	0.98	0.85	0.12	1.00	3	0.02	3.87
Romania	RO	4	191	0.52	0.20	0.06	0.95	2	0.48	3.75
Slovakia	SK	4	200	0.24	0.21	0.16	0.62	1	0.49	7.78
Slovenia	SLO	5	214	0.08	0.15	0.13	0.89	1	0.23	2.48
Spain	E	4	240	0.90	0.67	0.03	0.97	3	0.25	1.62
Sweden	S	3	176	0.30	0.48	0.17	0.21	3	0.26	0.56
Switzerland	CH	7	220	0.19	0.11	0.14	0.73	1	0.27	4.78
UK	GB	3	213	0.39	0.42	0.04	0.89	3	0.28	13.42

#### Social system variables

##### Regulation of clearcutting

National and sub-national forest policy and laws are key variables as they prescribe and regulate forest management, as well as serve as regulatory transmissions of international and EU policies. These on-the-ground rules set the foundations for what forest managers must do or can do in their forests in terms of timber harvesting, restocking, etc. Following Sotirov et al.^41^ there are three main types of countries with different national regulations for forest management used in Europe; (type 1:) countries with clearcutting without area size restrictions promoting CC, (type 2:) countries with clearcutting with area size restriction still allowing but constraining CC, and (type 3:) countries with clearcutting bans promoting the use of CCF and avoiding CC. Thus, presence of these types of national or subnational regulations provides a relevant social system indicator. The hypothesis is that we would expect a predominance of CC in type 1 countries, a predominance in CCF in type 3 countries, and a mixture of (more) CC and (less) CCF in type 2 countries.

##### Land ownership being public or private

There is increasing awareness that forest ownership plays an important role for the portfolios of barriers and bridges associated with the implementation of forest management policy on the ground. For example, interactions between ownership type, forest management approach and policy, are of fundamental importance. These interactions can be clustered in several distinct decision-making rationality groups that are present, in different proportions, in all European countries as indicated by synthesis and review studies^65–68^. In particular, group 1 of large private or industrial forest ownership usually applies intensive forestry such as CC for maximum timber production to meet industry and market demands and make revenues (“homo economicus”). On the contrary, group 2 of small scaled non-industrial private forest ownership usually applies partial cutting for sporadic household needs (“homo sociologicus”) or does not use timber at all due to urban and environmental lifestyle (“homo psychologicus”), both supporting CCF. In between, group 3 of public forest ownership usually applies medium intensive forestry combining both CC and CCF to meet diverse societal needs and policy objectives (“homo sociologicus” and “homo economicus”^65,67^. Empirically, Deuffic et al.^65^ identified five main forest owner categories in European countries, namely economic-oriented, tradition-oriented, environmentalist, non-active and multi-objective, but the distribution among countries is still tentative^41^. We hence used as a proxy for the diversity of forest owner groups the data about forest ownership in Europe compiled by Pulla et al.^69^ and UNECE^70^.

##### Jobs by forest area

Job creation remains the foremost concern for most countries, and forests are viewed as an important component of employment generation efforts, especially in rural areas^71^. The number of jobs derived from forest management and forest industry vary in both time and space, and is an important social system indicator. Ultimately, large numbers of available jobs relative to the forest area offers opportunity to using multiple goods, services and values, and thus implicitly to apply a diversity of forest management systems. We used the monitoring of forest and wood-based employment dataset for the European Union^72^ and combined this with forest cover data for 2015 from Forest Europe^73^ into the ratio forest jobs per 100 ha (Table 1). We added forest and wood-based employment data for Switzerland^74^ and Norway (https://www.regjeringen.no/en/topics/food-fisheries-and-agriculture/skogbruk/innsikt/skogbruk/id2009516/).

### Data analysis and results

The exploratory analyses were made in four steps. First, we compiled parameter values for the nine indicators listed in Table 1 in ArcGIS Pro and Microsoft Excel, and made descriptive statistics focusing on correlations among the nine variables. Second, we made nine thematic maps showing the spatial distribution of different parameter values for each of the nine variables across 26 countries in Europe. Third, using Past statistics^75^ we (a) standardised the data as a primary step to have normally distributed data and reduce the variance of the variables for further analysis, (b) performed a multivariate principal component analysis (PCA) using a variance-covariance matrix, (c) followed by a cluster analysis using Ward’s method. We then used both non-parametric and parametric statistics to explore the extent to which the Principal Component variables could explain the variation in the application CC and CCF forest management systems among countries using a rank index of 1–7 derived from Mason et al.^42^, see Fig. 4. The parameter values for biophysical, anthropogenic and social indicators are shown in Table 2. Significant Spearman rank correlations for individual variables are shown in Table 3. The strongest correlations (*p* < 0.01) were found between Cur intol and Slope, Con gain and Veg per, Cur intol and Nat intol, Wood harv and Nat intol, and Jobs/100 ha and Con gain, respectively. The nine maps presenting raw not standardized parameter values for the nine indicators show the large variation across 26 European countries (Fig. 1).

**Table 3:** Spearman rank correlation values for the relationships between the nine variables in Table 1 are given in the lower triangle of the matrix, and the two-tailed probabilities that the columns are uncorrelated are given in the upper triangle; p-values < 0.05 are underlined.

	Slope	Veg per	Nat intol	Cur intol	Wood harv	Con gain	CC rules	State forest	Jobs / 100 ha
Slope		0.07	0.73	*0.01*	0.62	0.70	0.07	0.09	0.42
Veg per	0.36		0.17	0.54	0.09	*0.00*	0.75	0.63	0.08
Nat intol	0.07	0.28		*0.01*	*0.00*	0.39	0.50	0.75	0.17
Cur intol	−0.47	−0.13	0.50		0.58	0.33	0.18	0.84	0.07
Wood harv	−0.10	−0.34	−0.61	−0.11		0.16	0.97	0.52	0.80
Con gain	−0.08	0.61	0.18	−0.20	−0.29		0.12	0.55	*0.00*
CC rules	−0.36	0.07	0.14	0.27	0.01	0.31		0.25	0.75
State forest	−0.34	−0.10	0.07	−0.04	−0.13	0.12	−0.23		0.36
Jobs / 100 ha	−0.17	0.35	−0.28	−0.36	−0.05	0.57	−0.07	0.19	

**Fig. 2:**
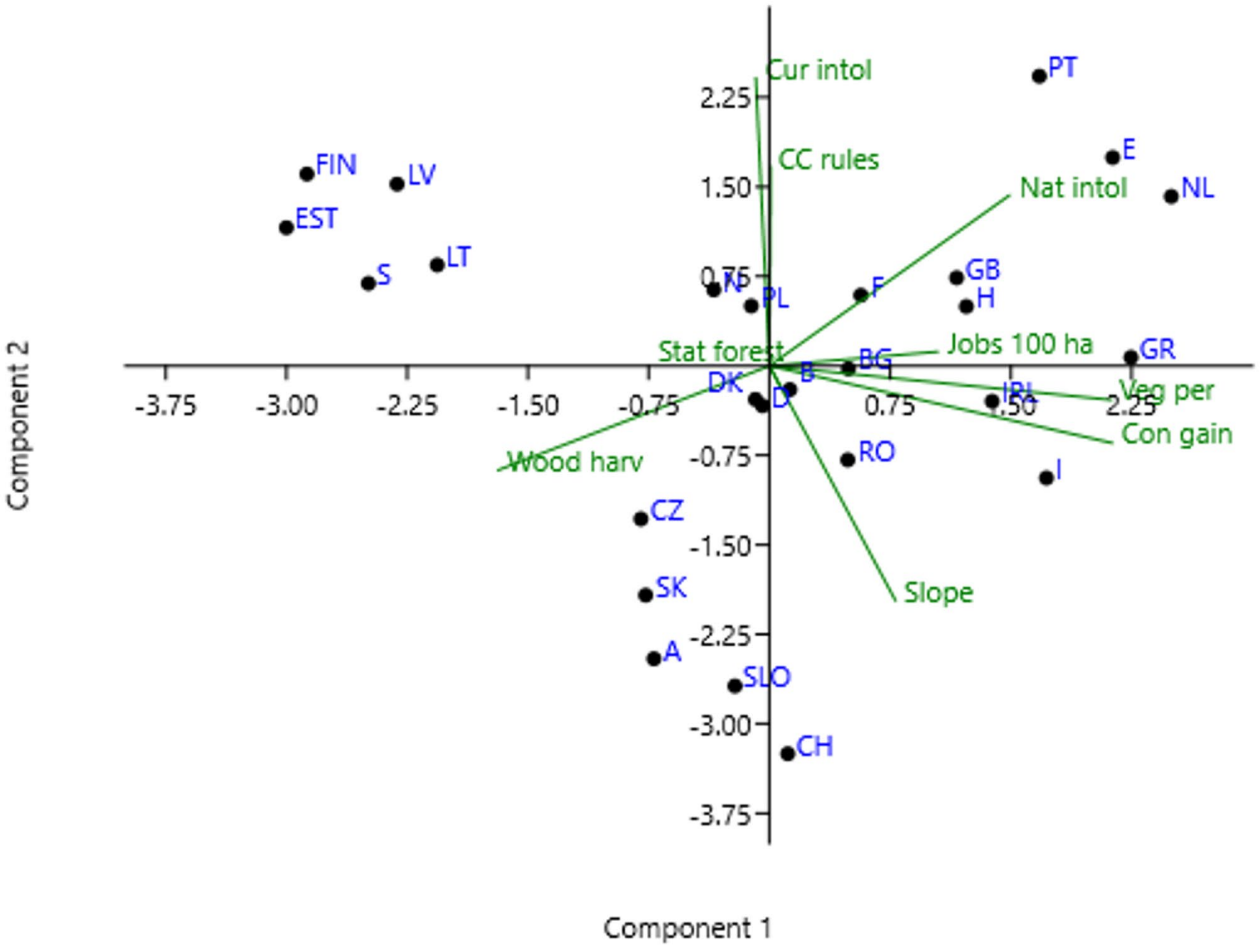
Results from Principal Component Analysis of nine standardised variables representing 26 countries in Europe.

In the multivariate principal component analysis’ (PCA) (Fig. 2), the first three components explained 66% of the variation in the dataset, with PC1 (27%) being positively related to coniferization and vegetation period, and PC2 (23%) positively related to current shade intolerant tree species and negatively related to terrain slope (Tables 4 and 5). PC3 explained an additional 16%, which was positively related to jobs per forest area, and the proportion of public forest.

**Table 4:** Summary result values from the PCA.

PC	Eigenvalue	% variance
1	2.49	27.64
2	2.05	22.73
3	1.45	16.16
4	1.27	14.07
5	0.67	7.45
6	0.58	6.47
7	0.23	2.52
8	0.13	1.49
9	0.13	1.46

**Table 5:** PCA loading values.

	PC 1	PC 2	PC 3	PC 4	PC 5	PC 6	PC 7	PC 8	PC 9
Slope	0.20	−0.49	−0.47	−0.08	−0.06	0.04	−0.04	0.57	0.39
Veg per	0.53	−0.07	−0.08	0.25	−0.08	0.39	−0.63	−0.22	−0.21
Nat intol	0.37	0.36	−0.29	−0.38	−0.03	0.10	0.32	0.28	−0.56
Cur intol	−0.02	0.61	−0.13	−0.07	−0.35	0.40	0.01	−0.04	0.58
Wood harv	−0.42	−0.22	−0.03	0.33	−0.06	0.70	0.29	0.13	−0.27
Con gain	0.54	−0.16	0.13	0.16	0.30	0.15	0.59	−0.32	0.28
CC rules	0.00	0.42	−0.09	0.53	0.60	−0.07	−0.10	0.39	0.05
State forest	0.02	−0.04	0.56	−0.51	0.40	0.37	−0.20	0.27	0.11
Jobs / 100 ha	0.26	0.03	0.57	0.32	−0.51	−0.14	0.11	0.45	−0.03

**Fig. 3:**
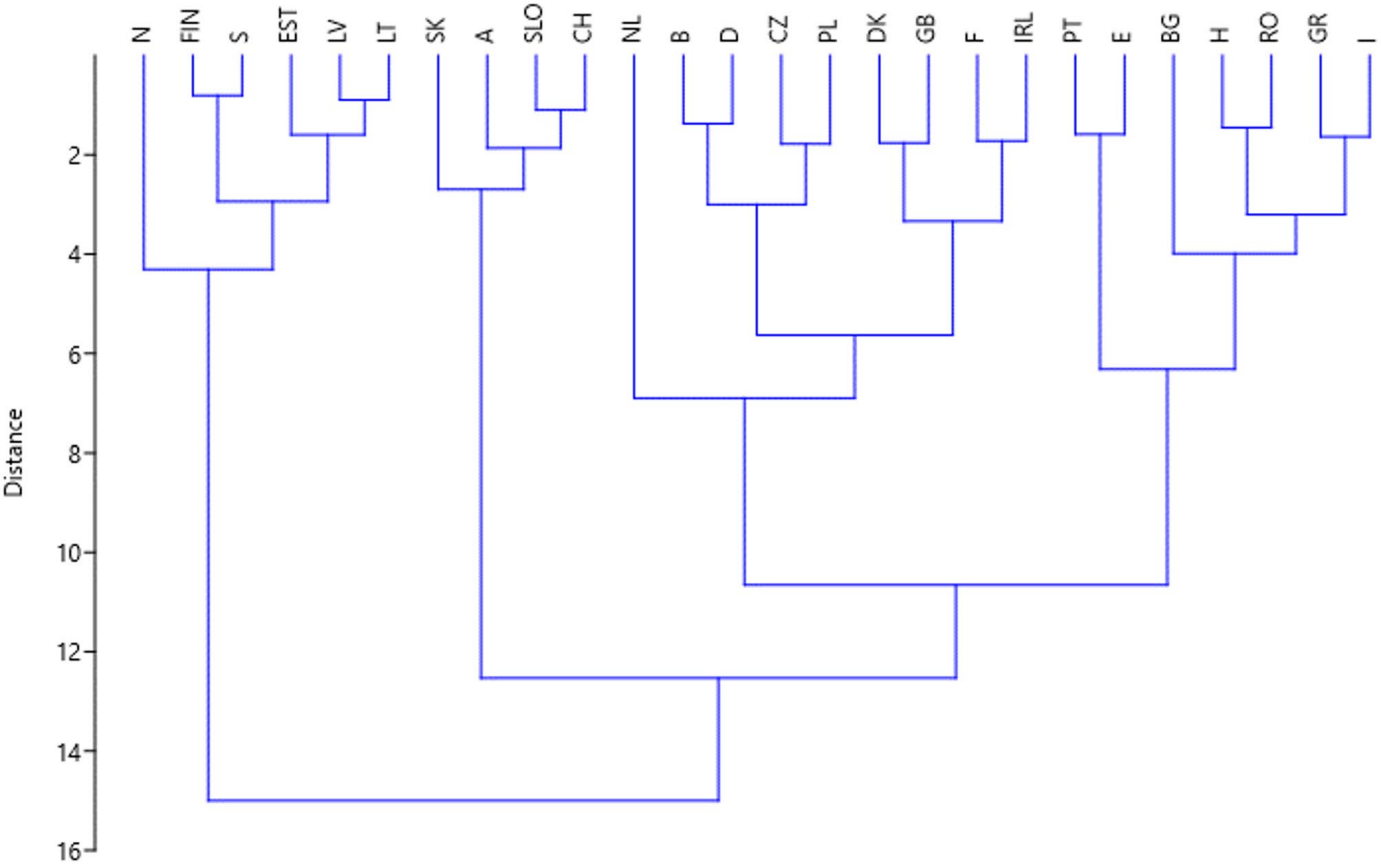
Results of the cluster analysis. The cluster analysis identified 4 main clusters; from left to right these represent (1) Nordic-Baltic countries with boreal forests, (2) central European countries with mountainous forests, (3) west and central European countries with lowland temperate forests, and (4) Mediterranean and SE European countries.

The PCA (Fig. 2) and cluster analysis (Fig. 3) reveal distinct patterns among European countries based on forest-related biophysical, anthropogenic and social system variables. Four regional groups were identified. (1) Nordic-Baltic countries with boreal and boreo-nemoral forests (e.g., Norway, Sweden, Finland, Estonia, Latvia, Lithuania), primarily positioned on the production-focused end of the forest management spectrum. The PCA shows that these countries exhibit lower vegetation cover and conservation gain scores, coupled with high levels of wood harvesting. They are also moderate in environmental regulation intensity, reflecting a long-standing reliance on intensive even-aged forestry and well-developed wood industries rooted in boreal forest ecosystems. (2) Central European countries with mountainous forests (e.g., Austria, Switzerland, Slovakia, Slovenia), characterized by balanced multifunctional forestry, combining moderate production, strong conservation gains, and higher levels of governance and regulation, including sensitivity to nature protection concerns. Their position on the PCA reflects a middle ground between production and protection, aligned with traditional mountain forestry values and EU-aligned environmental standards. (3) Western and Central European countries with lowland temperate forests (e.g., Netherlands, Belgium, Germany, Czech Republic, Poland, Denmark, Great Britain, France and Ireland) exhibit intermediate to high vegetation cover and conservation gains, paired with moderate levels of wood harvesting. Their high scores on PC2 reflect strong environmental regulation and governance structures. These countries align most closely with multi-functional forest management models, where wood production is balanced with considerations towards biodiversity conservation and societal preferences for ecosystem services. (4) Mediterranean and Southeastern European countries dominated by deciduous trees (e.g., Portugal, Spain, Bulgaria, Hungary, Romania, Greece and Italy) were characterized by lower vegetation cover and conservation investment, along with relatively permissive governance environments for land-use intensification. Their positioning in the PCA suggests greater vulnerability to forest degradation, and lower emphasis on ecosystem-based management.

**Fig. 4:**
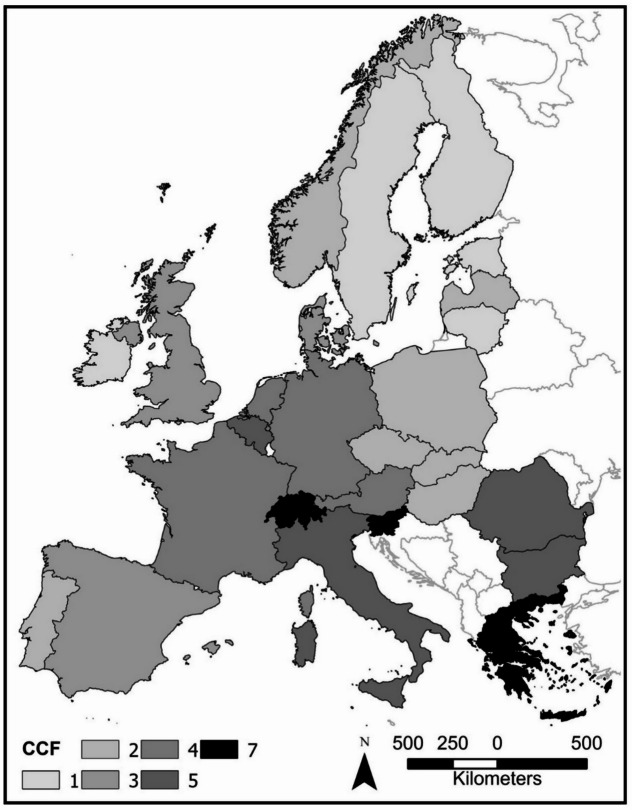
Map of Europe indicating the relative use of continuous cover forestry (rank values 1 to 7 were derived from Mason et al.^42^). The darker the shade of the country the higher the use of continuous cover forestry (CCF). The darker the shade of the country the higher the use of continuous cover forestry (CCF). The map was created using ArcGIS PRO 3.4.0 (https://www.esri.com/en-us/arcgis/products/arcgis-pro/overview).

**Fig. 5:**
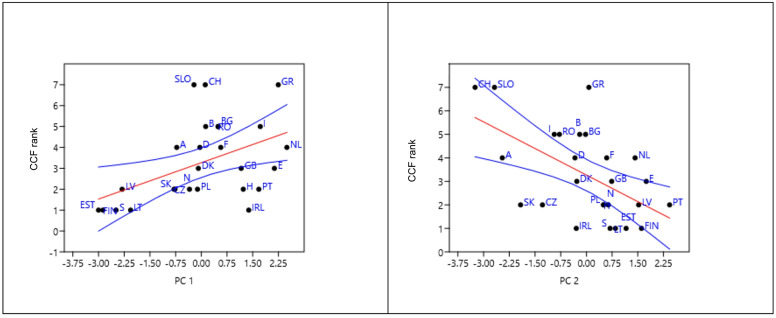
Scatter plots of relationships among 26 countries between a CCF index based on Mason et al.^42^ and PC 1 (left) and PC 2 (right). The CCF index scale ranges from 1 to 7, where 1 represents no use of CCF methods in a country, and 7 represents countries where only CCF is used.

We then explored the hypothesis that the application of CC and CCF management systems, respectively, can be explained by the portfolio of biophysical, anthropogenic and social system variables. There was large variation in the application of CC vs. CCF forest management approaches in Europe (Fig. 4). Both PC1 and PC2 were correlated to the rank index derived from the proportions of CCF used in the 26 countries (Fig. 5). Spearman r rank two-tailed tests were used for comparison of CCF rank index and PC 1 yielded r-value of 0.51 (*p* = 0.008) and for PC 2 an r-value of −0.51 (*p* = 0.007), while PC 3 not being statistically significant with *r*=−0.07 (*p* = 0.73). A multiple regression including PC1 and PC2 had an R-square value of 0.53.

## Qualitative analysis

### Regional distribution of CC and CCF use in Europe

We stress the tentativeness of this study for two groups of reasons. First, there are multiple variables that potentially may represent biophysical, anthropogenic and social system drivers^44^, and a different selection may yield other patterns. Second, countries are large and exhibit considerable internal variation due to latitude (such as Finland, Norway and Sweden) and altitude (countries with mountains). Summarising parameter values for an entire country in one value is thus a considerable simplification. The analysis would be more robust if based on a more regional level with smaller geographical units and having more datasets. However, coarse country-level data about the use of CC and CCF^42^ is what is readily available based on a consistent classification approach. Importantly, even with limited statistical precision, given the aim of this exploratory study, the accuracy is still sufficient for not rejecting the pattern that a suite of biophysical, anthropogenic and social system variables matches the relative occurrence of CC and CCF forest management methods in Europe.

Given these caveats, this macro-level exploratory study with countries as focal units illustrates that the distribution of CC and CCF forest management systems across Europe are associated to a suite of biophysical, anthropogenic and social system factors. The biophysical variables Slope (once), Veg per (once) and Nat intol (twice) were involved in statistically significant relationships, as well as the anthropogenic variables Cur intol (twice), Con gain (twice) and Wood harv (once), and the social variable Jobs/100 ha (once) (see Table 3).

The clustering of countries using the multivariate analyses (Fig. 3) matches the four biogeographical regions presented by EU Habitats Directive Art 16 reporting, i.e. boreal, mountainous, temperate, and Mediterranean^76^. Regarding the conservation status of forests in these four groups of countries, the boreal forests stick out with much higher proportion with unfavourable conservation status (64%) compared to the other three regions (from 16 to 28%). The same pattern regarding conservation status (45% vs. 12–21%) among these forest regions has been reported in the impact assessment report by the European Commission^76^. The main pressure behind this pattern is related to high forest management intensity in boreal forests (e.g., clearcutting, short rotation, loss of natural and old-growth forests, and afforestation with non-native species such as *Pinus contorta*). This has bearings also on social system variables, which is indicated by the inferior states and trends of boreal forests coinciding with the lowest number of jobs/100 ha, which is linked to intensive forest management (Robert et al. 2020), in the boreal forest countries (1.3 jobs/100 ha) compared to the other three groups (3.1, 4.5 and 7.7 jobs/100 ha) (data in Table 2, Kruskal-Wallis test for equal medians 12.1, *p* = 0.005)).

Comparing 27 countries in Europe, Nagel et al.^77^ also observed clear differences among regions with respect to management approaches. They found that countries in Central (temperate forest) and Northern (boreal forest) Europe prioritize intensive timber production based on using even-aged, rotational management (CC). Countries in the south-eastern part of the temperate, mountainous and Mediterranean forest regions mostly use extensive management with uneven-aged and continuous cover methods.

Additionally, in the Mediterranean, cultural woodland management is traditional and not focused of sustained yield of wood as CC and CCF methods are. In the Mediterranean region, a high degree of resilience resulted in a dynamic coexistence of integrated social-ecological systems^78^. Blondel^78^ used the term design of landscapes metaphorically to indicate the long-lasting influence of human impacts, which resulted in the unintentional shaping of a multifunctional cultural landscapes. Regarding forests, Nocentini’s et al.^79^ review of current trends shows that wood production is a main contemporary forest management objective, but with an increased focus on non-wood forest products. At the local level impacts of management for coppice vs. high forest, and abandonment of cultural woodlands were reported as drivers, while climate change impacts and disturbances were considered crucial at the regional level. Wildfire is also a current key issue, and Bergmeier et al.^80^ highlight that abandonment of traditional multiple use management enhances encroaching woody plants in cultural woodlands, and afforestation with conifers, are leading to increasing amounts of combustible biomass.

### Missing elements of CC and CCF

Both CC and CCF are challenged by delivering more than wood and biomass. A key topic is to understand the effects on conservation of viable populations of naturally occurring species, functional habitat networks and ecological resilience. It is thus insufficient to only consider the forest harvesting and management system and not its harvesting intensity and spatial pattern. Five examples are: rotation length, the age variation of trees within and among stands in landscapes and regions, tree species composition, size of treatment area, and harvesting intensity.

First, *rotation length* is a key component of both CC and CCF forest management systems. There are very large differences between the age class distribution in managed and naturally dynamic forests^81,82^. Using Fennoscandian forestry as a case, Roberge et al.^83^ reviewed implications for both ecological and social systems of modifying rotation lengths^84^ relative to current practice. Shortened rotations were expected to be mostly negative to neutral (e.g., production of wood, bilberry, reindeer forage), and can limit damages such as root rot and bark beetles. Other damage types may increase and impede climate mitigation. Supporting and cultural ecosystem services would be affected negatively by shortened rotations, and positively by extended rotations, as would most biodiversity indicators. In northern Sweden there is an ongoing transformation of forests originating from long rotations (160–180 years^13^ and selective fellings of large trees, to shorter even-aged rotations^85^. Eggers et al.^86^ showed that business-as-usual clearcutting even-aged forest management would extend the past seven decades of ground lichen habitat decline by an additional 50% during the next 50 years. It should be noted that lichen habitat is already recognized as critically low by reindeer herders.

Second, it is crucial to pay attention to the *age distributions* of both trees in stands, and of stands in landscapes. In even-aged forest management tree age and stand age are strongly correlated, and stand age distributions in managedt landscapes are typically bimodal. The dominating age classes are evenly distributed until the final felling age, and a small proportion of older forest is set aside as isolated patches. In uneven-aged forest management stand age can be estimated as the time after the most severe recent disturbance. However, there are both younger and older trees, the latter providing opportunity for harvesting larger and more profitable tree individuals. However, in both examples sufficient amounts of habitat structures such as dead standing trees, snags and downed wood in different stages of decomposition need to be maintained at the landscape scale. There is consistent empirical knowledge for this conclusion from studies of a wide range of taxa^87^. For example, a study of lichen species on downed deadwood Hämäläinen et al.^88^ showed that the lichen density increased with the amount of > 100 years old stands in the surrounding landscape. Effective management for lichen species dependent on deadwood thus requires securing habitat conservation at the landscape scale. This is consistent with a comparison of even-aged and uneven-aged forest management in Central European beech forests^89^. Their study showed that a mosaic of different age classes was more important for regional biodiversity than high within-stand heterogeneity and therefore suggested reconsidering the current trend of replacing even-aged management in temperate forests. Instead, the variability of development stages and stand structures should be increased to promote landscape-scale biodiversity, provided that sufficient areas of unmanaged forests are present on the landscape. This is the reason that transformations of landscapes dominated by structurally rich successional stages, both earlier and later^90^, to shorter rotations result in loss of habitat for species that depend on high levels of naturalness^82^.

Third, transitions from natural and historical ranges of variability involve *tree species* transitions. A focus on developing cropping systems aimed at conifers have reduced the amount of mixed and broadleaf forests. Lindbladh et al.^62^ studied the spatial and temporal patterns and processes underlying the dominance of Norway spruce in southern Sweden. Combining paleo-ecological methods to document long-term changes, and National Forest Inventory data to document short-term changes, they showed that spruce was widespread and abundant already 1000 years ago. After a small decline around 500 years BP, there was a rapid increase from 1920 to 1950. This was linked to abandonment of forest grazing, and continuous cover forestry benefiting spruce regeneration. Subsequently, industrialized forestry began in the 1950 s, which increased the amount spruce even further.

Fourth, the *size of treatment areas* affects the grain size of forest landscapes’ constituent patches of different development stages. Naturally dynamic forest landscapes include a mixture of patch sizes and different development stages. Because species have different minimum patch size requirements, the grain size affects the distribution of species. A coarse-grained landscape with rotation times of, say, 100 years is able to host the full suite of European forest grouse species depending on young, middle-aged and old forest, respectively^91^. Good abilities to habitat tracking means that the shifting mosaic of sufficiently large patches of different development stages will sustain such species in the longer term^92,93^. However, such managed landscapes have poor capacity to host species with high requirements of naturalness.

Fifth, *harvesting intensity* is a key factor affecting habitat structure irrespectively of forest management system. This applies to both total growth on forest land available for wood supply, and the standing volume of any residual living habitat trees, snags and dead wood in stands subject to final felling or commercial thinning. There are empirical, though scant, data about the necessary amount of retention structures compared to the amount in unharvested stands. This ranges from a minimum of 5–10%^94^. Aubry et al.^95^ suggested that in the US Pacific Northwest retention levels > 15% are needed to effectively retain species, sustain microclimatic conditions and gain public acceptance. They concluded that a combination of setting aside 1-ha aggregates plus retention trees at this level, being considerably greater than current minimum standards, is an effective strategy. Evaluating retention forestry 10 years after its introduction in temperate forests, Großmann et al.^96^ analysed the resulting provision of tree-related microhabitats and dead wood. In contrast to common retention levels of < 5%, they showed that a conversion of 15–25% of the stand area into habitat tree groups is needed to exceed minimum thresholds for the stand level abundance of tree-related microhabitats such as cavities, exposed sapwood, or crown dead wood significantly in the short term.

**Fig. 6:**
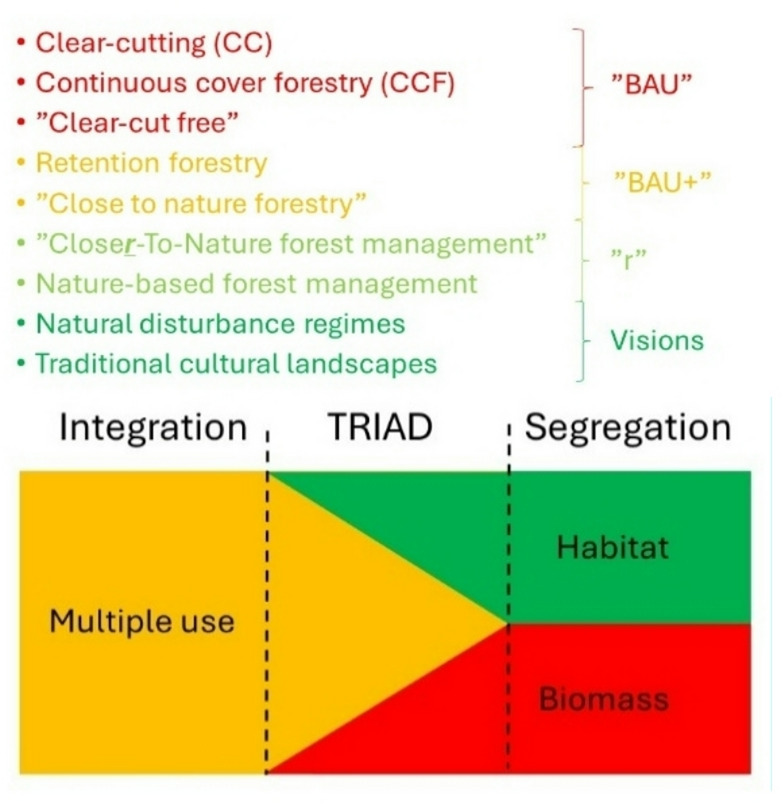
Forest management systems in Europe range from those mainly focusing on wood production (red), those that aim at delivering multiple benefits such as wood, biodiversity, carbon and other ecosystem goods and services (orange), and others that mainly aim at biodiversity conservation by emulating natural habitat dynamics, ecosystem integrity and resilience (green). To really succeed with all these ambitions requires application of several forest management systems ranging from traditional clear-cutting (CC) and continuous cover forestry (CCF) to nature-based closer-to-nature forest management. Additionally, spatial planning of landscapes that matches the interests and opportunities among different forest owner and land user categories is needed. Natural disturbance regimes and traditional cultural landscapes are two complementary visions.

To conclude, these five missing or poorly covered elements listed as deficiencies of CC and CCF require transitions towards nature-based forest management (Table 6; Fig. 6). The following section outlines how. Both CC and CCF forest management are indeed good at sustained wood and biomass production. However, the emergence of policy aimed at biodiversity conservation^36,97^ thus calls for additional novel forest management approaches. The reason is that both CC and CCF approaches result in reduction of forest stand variability^22,98,99^, and thus reduce the level of naturalness with structures at different spatial scales like old trees and tree-related microhabitats^100,101^, dead wood in different decays stages^102^, and intact forest landscapes^85^. Therefore, neither CC nor CCF forest management systems are well suited at maintaining biodiversity in terms of naturally occurring species, the habitats they require, and the processes that maintain them.

**Table 6:** The large number of concepts to describe different forest management systems can be divided into four different groups. The first represents the focus on the production of timber and pulpwood (Business as usual (BAU); the second the addition of nature considerations (BAU+), the third to come even closer to nature, and the fourth represents visions to strive towards and which reflects different regional contexts in Europe. The “traffic light approach” reflects duncker’s et al.^127^ classification of different forest management systems based on how much they alter natural processes and structures.

Label	Forest management intensity	Forest management systems	Description	References
BAU	Very high	Plantation forestry	Fast-growing biomass	McEwan et al.^139^
BAU	High	Even-aged rotation forestry	Clearcutting system, 1–2 species	Angelstam et al.^3^
BAU	High	Continuous cover forestry	Selection system	Mason et al.^42^
BAU	High	Shelterwood	Tradition in continental Europe	Raymond et al.^114^
BAU	High	Clearcut free	Response to critique against clear-cutting	Appelqvist and Mogren^16^
BAU+	Medium	Selective systems, ”wood mining”, high-grading	First wave to harvest large trees in old-growth forests	Lie et al.^140^, Naumov et al.^141^
BAU+	Medium	Retention forestry, “New forestry”	Aim to create better conditions for species	Fries et al.^142^, Gustafsson et al.^94^
BAU+	Medium	“Close-to-nature forestry”	Aim to create better conditions for multiple use	www.prosilva.org
“r”	Low	“Close**r**-to-nature forest management”	A new concept that pleads for more naturalness	European Commission^32,35^, Larsen et al.^4^
“r”	Low	Nature-based forestry, ecological forestry	Argues for more nature considerations and emulation of disturbances	Batavia and Nelson^143^, Palik and D’Amato^144^
“r”	Very low	Protected areas and voluntary set asides	Strict protection and active management	European Commission^36^
Vision	Reference system	Cultural landscape (grazing, mowing)	Vision for biocultural values	Vos and Meekes^145^
Vision	Reference system	Emulate natural disturbance regimes	Vision for species, habitat and resilience	Kuuluvainen et al.^39^

### Moving beyond CC and CCF

Traditional forest management approaches for high yield wood production have come under the microscope in recent years^15,103^. The failure of traditional classical forest management systems focusing of sustained yield to deliver multifunctional landscapes^104^ form the background of the EU’s New EU Forest Strategy for 2030^32^. This is illustrated by unfavourable status of European forests^105^, which is linked to over-regulation towards growth and yield that diminishes resilience to environmental stress as well as threatens biodiversity^106^.

As a complement to CC and CCF, the term “Closer-to-Nature Forest Management^4,35^ has been proposed. The small letter “r” in the first word of the term indicates a direction with a vision that is focused on learning towards multifunctional forest landscape management. As an example, EU’s new forest strategy for 2030 states that “*the supply of wood products should be done in synergy with improving the conservation status of European and global forests*,* and preserving and restoring biodiversity for forest resilience*,* climate adaptation and forest multifunctionality*”. In short, this approach aims at emulating the composition, structure and function of naturally dynamic forest ecosystems to which species have evolved, and traditional cultural landscapes (such as wooded grasslands), on a sufficiently large proportion of landscapes. These methods must be adapted to biophysical conditions and social system traditions in different European regions^4,42,107^.

To conclude, there are new demands on what forest management system should deliver. Thus, different traditional forest management approaches like CC and CCF represent Business As Usual (BAU) (Table 6; Fig. 6). Selective systems, retention forestry and “close-to-nature” forestry (BAU+) expands the characteristics of effective cropping systems by adding management aimed at increasing the amount of nature considerations. Further forest policy development within the EU has led to adding the small “r” in the term closer-to-nature forest management. This is consistent with nature-based or ecological forestry, which aims at biodiversity conservation and maintaining the resilience of complex forest ecosystems (Table 6). This includes sustaining habitat of sufficient quality and quantity so that viable populations of naturally occurring species are maintained, and recreation and outdoor life is supported, as well as encouraging value chains that build on those (Larsen et al. 2022, Muys et al. 2022).

## Discussion

### CC and CCF forest management systems are not enough

Implementing policies about multifunctional forest landscapes requires a social-ecological perspective, and recognition of both spatial and temporal features at multiple scales. However, while the history of studies focusing on sustained yield forestry in Europe cover centuries^3,108^, comparative studies about the effects of different forest management systems on different forest goods, values and services is short. In particular, conservation biology as an integrative discipline highlights that what landscapes should deliver is subject to cultural contexts and evolves over time^109^. In broad terms, European biodiversity conservation involves the maintenance of functional habitat networks at the landscape scale and represents visions of either forest naturalness^110^ or cultural woodland landscapes^111^. In a review of CC vs. CCF in Kuuluvainen et al.^112^ thus concluded that there “*remains a considerable gap in our knowledge regarding the ecological and economic outcomes and applicability of the two forest management alternatives*”. Comparisons of forest management systems with respect to the extent to which they align with sustainable forest management policy requires the use of different suites of response variables. We exemplify this by three topics not delivered if the focus is on biomass production: conserving naturally occurring species, multifunctional forests and social values (Table 7).

**Table 7:** Overview of studies using different response variable for comparing different forest management systems.

Response variable	Description	References
Naturally occurring species	Resident birds, lichens, saproxylic insects and fungi	Savilaakso et al.^17^ Brunet^117^ Nolet et al.^99^
Multifunctional forests	Soil fungal communities, recreational ecosystem services, climate change mitigation, habitat availability for vertebrates, and red-listed dead wood dependent species	Peura et al.^14^ Eyvindson et al.^120^ Kim et al.^123^
Social values	Aesthetics and recreation Cultural values Biocultural conservation	Holgen et al.^125^ Koivula et al.^126^ Rotherham^129^ Plieninger et al.^128^

Biophysical variables Anthropogenic variables Social system variables.

To conserve naturally occurring species, uneven-aged and mature even-aged forests (CCF) (> 80 years old) are more important compared to current shorter (60 to 80 years) rotations of even-aged forest management systems (CC)^17^. Nonetheless, protected and set-aside areas of natural forest remnants are needed to ensure conservation of forest dependent species and to succeed with biodiversity conservation. Thus, a mosaic of different forest management approaches must be combined within a forest landscape. This is supported by the objectives in both global conventions and EU policies about forest set asides and protected areas^31,32,36,113^. Furthermore, aiming towards closer-to-nature forest management through the emulation of natural disturbance patterns and processes is not a completely new strategy. In the late 19th century Gayer developed the “Femelschlag” forest management approach in Germany^114,115^. It applied a reproduction technique to produce mixed species forest stands by incorporating and encouraging broad-leaved and mixed deciduous and coniferous shade intolerant trees species, through the emulation of natural disturbance patterns (gap size) with multiple-age classes, thereby enhancing ecosystem complexity and resilience^108,116^. The Femelschlag approach is associated to the irregular shelterwood system. Brunet^117^ concluded that irregular shelterwood and selection system with varied size of gaps could provide favourable conditions for biodiversity conservation. However, to succeed with the conservation of viable populations of naturally occurring species, also requires nature considerations in terms of high-quality retention trees^118^ and large volumes of dead wood in different stages of decay amounting to 20–50 m^3^/ha at the landscape level^119^.

Regarding within-stand multifunctionality, Peura et al.^14^ showed that this was higher in continuous cover forests than in even-aged rotation forests. Therefore, continuous cover forests (CCF) may have a greater potential to produce simultaneous multiple benefits from forests. However, Peura et al.^14^ also duly noted that unmanaged forests often provided the highest levels of ecosystem services and biodiversity. This means that the role of unmanaged forests for delivering forest related ecosystem services, and biodiversity, is crucial. They concluded that CCF does not guarantee the maintenance of all ecosystem services and biodiversity in commercial forests, but it can be an important part of a successful progression towards more sustainable forest management. Eyvindson et al.^120^ concluded that if there is a requirement for high economic benefits, banning rotation forestry based on clearcutting to create even-aged forests does not promote forest biodiversity and multifunctionality at the landscape scale. However, they recommended CCF as a primary management alternative to clearcutting, combined at the landscape scale with the application of rotation forestry. This is consistent with a triad approach^121,122^. Kim et al.^123^ assessed the impact of CCF and clearcutting on soil fungi and chemical properties within forests dominated by Norway spruce. They found that fruiting body and soil fungal communities were broadly similar in CCF and unmanaged forest, but different in clearcut areas. For these taxa associated with unmanaged forest and mimicking natural disturbance regimes, their findings illustrate that CCF is an alternative compared to CC.

Social values are a third motive for choosing a forest management system. This includes cultural ecosystem services like aesthetic and recreational values, which are attractive to people^124,125^. Koivula et al.^126^ used photo surveys to study Finnish citizens’ views of attractiveness of within Scots pine (*Pinus sylvestris*) stands subject to no harvest, CCF, seed-tree shelterwood and CC, a gradient along which attractiveness declined. The respondents’ gender, education, living environment, memberships in recreational or nature NGOs, forestry profession, and forest ownership had negligible effects. They thus recommended the use of CCF in settlement and recreational areas. Duncker et al.^127^ defined a gradient in management intensity among forest management approaches and forest ownership from high to low intensity based on how much they alter natural patterns and processes. This has led to multiple efforts to define forest ownership types based on desired goods, values and services^66,68^. As a final example about coping with social values, Plieninger et al.^128^ stressed that cultural landscapes in the Mediterranean represent social-ecological approaches, which require maintenance of traditional and diverse land-use systems. Similarly, Rotherham^129^ focuses on ancient woods and forest landscapes in the UK and notes that cultural knowledge and memories associated to traditional management is reduced. Therefore, the cultural heritage of these woods and landscapes is threatened. Cultural severance is a major driver of ecological change and species loss. This requires coping with a mix of values, rules, and knowledge in decision-making, an issue also applicable to CC and CCF.

### Traditional and new management systems, and landscape planning

There are increasing expectations that forests should be sustained as multifunctional landscapes by providing a wide range of goods, services and values. However, no single management or planning system can deliver all desired benefits that are desired (Fig. 6). While traditional CC and CCF systems for forest management are effective at producing wood and biomass, they are less effective in supporting biodiversity conservation and adaptive capacity^130^. Maintenance of forest ecosystems is thus a concern, and therefore also revolves around the long-term persistence and resilience of ecosystem components, structures and functions. Thus, a diversity of nature-based forest management approaches also needs to be applied. The concept “closer-to-nature forest management” is thus a complement to CC and CCF, which aims at pushing forest management systems towards higher levels of naturalness^4^. While Wang et al.^131^ did not mention “closer-to-nature forest management” explicitly, they made a call for a global commitment to rewilding-inspired forestry with the same explicit focus on nature-based forest management. However, in a recent detailed mapping of forest management systems in Europe, Scherpenhuijzen et al.^132^ did not include the need for nature-based management as defined in closer-to-nature forest management. This illustrates the challenge of terminological nuances. In spite of the similarity in wording there is a fundamental difference between “close to nature forestry” and “closer-to-nature forest management”. While the first is in principle the same as CCF, the second is about nature-based forest management – inspired by both natural disturbance regimes and by the traditional management of cultural landscapes. Figure 6 illustrates this.

Using the boreal-temperate transition in Europe as a case study, Manton et al.^57^ identified several mismatches between current forest management systems and naturally dynamic forest ecosystems. Such mismatches form obstacles for developing closer-to-nature forest management. To explore mismatches, they (i) quantified natural representative and contemporary forest types, (ii) matched current vegetation types with their predicted natural disturbance regimes, (iii) analysed modification of tree species composition, (iv) compared natural tree life expectancy with harvest age, and (v) compared current stand age distributions with those of predicted natural disturbance regimes. Results demonstrated clear mismatches between policy visions and current practices. Coping with mismatches between policy advocating multifunctional resilient forest landscapes that are sufficiently similar to naturally dynamic forests on the one hand, and current forest management systems focusing on wood production on the other, requires multiple solutions. This includes not only application of closer-to nature forest management approaches that can emulate natural disturbance regimes at tree and stand scales^39^, but also landscape planning and multi-level governance approaches.

Thus, the tripartite TRIAD approach including conservation, multiple-use, and wood production^122^ is a valid solution^77,121,133–135^. Nevertheless, as shown by Nagel et al.^77^ zoning in Europe is overwhelmingly focused on wood production, but with little concomitant protection of forests in strict reserves. Additionally, most strict forest reserves are < 50 ha in size, likely too small to capture the minimum dynamic area necessary to sustain habitat for many specialised species. We argue that this merits this paper’s focus on forest management systems and how they can be modified. However, depending on landowners’ preferences, collaborative capacity and available policy instruments, the opportunities for this vary considerably among local landscapes, regions and countries^103,136^. To avoid trade-offs at small spatial extents (individual forest stands), one can manage conflicting goals on larger spatial extents (forest management unit or estate, and the entire landscape) by deliberately doing different things in different areas^49^. Figure 6 summarises the combination of traditional and new approaches to forest management and zoning of forest landscapes at multiple scales from trees and stands to landscapes and regions.

Applying different portfolios of forest management systems and changing between them faces numerous challenges^137,138^. This involves long-term legacies about what is “right”, dynamic societal desires and macroeconomic drivers as well as biophysical disturbances including climate change impacts and need to satisfy ecological, economic, cultural and social sustainability. A key challenge for landscape planning of different forest management approaches is to cope with the complexity of stakeholder portfolios at multiple levels of governance.

## Data Availability

The datasets used and/or analysed during the current study is available from the corresponding author on reasonable request.
